# A Metasystem of Framework Model Organisms to Study Emergence of New Host-Microbe Adaptations

**DOI:** 10.1371/journal.pone.0003891

**Published:** 2008-12-10

**Authors:** Suresh Gopalan, Frederick M. Ausubel

**Affiliations:** 1 Department of Molecular Biology, Massachusetts General Hospital, Boston, Massachusetts, United States of America; 2 Department of Genetics, Harvard Medical School, Boston, Massachusetts, United States of America; Centre for DNA Fingerprinting and Diagnostics, India

## Abstract

An unintended consequence of global industrialization and associated societal rearrangements is new interactions of microbes and potential hosts (especially mammals and plants), providing an opportunity for the rapid emergence of host-microbe adaptation and eventual establishment of new microbe-related diseases. We describe a new model system comprising the model plant *Arabidopsis thaliana* and several microbes, each representing different modes of interaction, to study such “maladaptations”. The model microbes include human and agricultural pathogens and microbes that are commonly considered innocuous. The system has a large knowledge base corresponding to each component organism and is amenable to high-throughput automation assisted perturbation screens for identifying components that modulate host-pathogen interactions. This would aid in the study of emergence and progression of host-microbe maladaptations in a controlled environment.

## Introduction

The intermingling of organisms not previously in contact due to societal rearrangements has a huge potential to bring about new diseases and afflictions to human kind (directly and indirectly by affecting agricultural and other essential resources) due to adaptation of microbes into potential pathogens on new hosts. One mode of such adaptation would be a consequence of adaptive changes in system status (viz., rewiring of and crosstalk between pre-existing signaling components and networks and alterations in gene regulatory networks) of the host and/or the microbe under appropriate environmental conditions. Another example for the emergence of new pathogens includes some nosocomial infections. For instance, many bacterial and fungal opportunistic pathogens form biofilm on catheters and infect immunocompromised patients, where there is change in system status in both host and microbe that facilitates the interaction [1]. The adaptation of microbes to new hosts, the failure to eradicate them, and continued availability of appropriate host niche, could lead to permanent fixation through genetic changes over time. In the case of bacteria, the rapid acquisition of virulence or resistance to drugs could also occur through lateral gene transfer, e.g., on plasmids [2]. The existing models and studies of ecological and evolutionary principles of host-microbe interactions focus on the second stage of this adaptation (genetic fixation, e.g., genome wide association studies) and on microbial communities in hosts [3]–[6]. The latter case involves the study of complex adaptations of host and microbial communities in unison, as highlighted by recent studies on the human microbiome.

Studying the mechanistic basis of the emergence of new diseases through an intermediate adaptive stage is hard to study in natural settings. Thus, we have established a laboratory system using the model host plant Arabidopsis interacting with different microbes. The study of host-microbe interactions in this setting gives insight into how a host responds to a variety of microbes under controlled conditions and how so-called innocuous microbes can cause disease in certain circumstances. The data presented below demonstrate that this system encompass a variety of modes of host-microbe interactions, thus making it a powerful system to understand the fundamentals of this phenomenon.

## Results and Discussion

**Figure 1:**
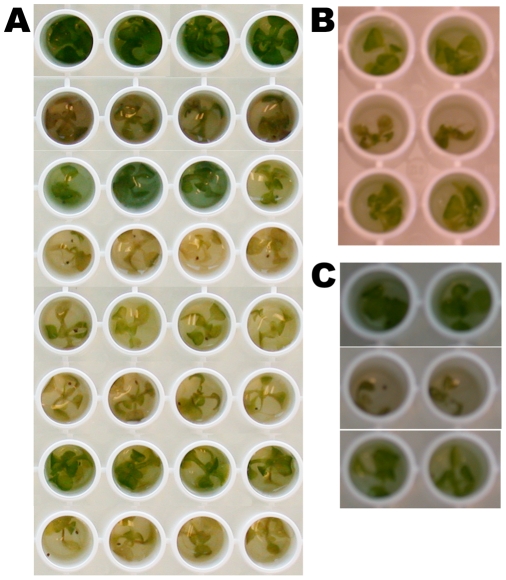
Visual phenotype of seedlings infected with different microbes. Seedlings grown in 96 well plates (one per well) were infected with indicated microbes and symptoms recorded (A) 5dpi, (B) 3dpi and (C) 4dpi. (A) the different rows display seedlings 1. untreated, or treated with 2. *P. aeruginosa*-PA14, 3. PA14::*lasR*, 4. *B. subtilis*, 5. *E. coli* (Dh5a), 6. DC3000, 7. DC3000::*hrcC*, 8. DC3000(pAvrB). (B) rows: top-untreated control, middle-*E.coli*, 3. bottom-*E.coli* expressing GFP in a plasmid. (C) rows: top-untreated control, middle-*B. subtilis*, bottom-*Staphylococcus aureus*.

When 10-day old Arabidopsis seedlings growing submerged in liquid plant growth medium in 96 well microtiter plates are infected with the plant pathogen *Pseudomonas syringae* pv. *tomato* (DC3000) or the multi-host opportunistic human pathogen *Pseudomonas aeruginosa* (PA14) the seedlings developed chlorotic disease symptoms and exhibited growth arrest (Fig. 1A). Surprisingly, the gram-negative and gram-positive laboratory microbes *Escherichia coli* and *Bacillus subtilis*, respectively, also inflicted damage and caused growth arrest of Arabidopsis seedlings under these conditions (Fig. 1A, Fig. 1B and Fig. 1C). The pathogenic behavior of DC3000 and PA14 was not surprising-PA14 had earlier been shown to infect a variety of invertebrate hosts, including plants, in addition to mammals [7]–[9]. But the fact that laboratory microbes generally considered to be nonpathogenic can affect seedlings similarly to known pathogens was not expected and provided an opportunity to study host-microbe maladaptations in a controlled environment. In particular, *B. subtilis* was especially potent in inflicting damage, such that in three days the seedlings were quite bleached and there was a significant loss of tissue integrity (data not shown and Fig. 1A). Importantly, under the conditions of these assays, the microbes do not grow significantly in the plant growth medium, but grow well in the presence of seedlings, indicating that active host-microbe interaction contributes to microbial growth and host damage (Table 1 and [10]). In contrast to submerged seedlings, 10 day old seedlings growing on soil were not susceptible when sprayed with *B. subtilis* or *E. coli*, whereas DC3000 caused potent chlorosis and stunting (Fig. S1 and data not shown). Unlike seedlings, leaves of adult Arabidopsis plants infiltrated with *B. subtilis* did show tissue damage limited to the infiltrated area. This damage was characteristically different, confined (Fig. S2) and slower compared to the typical spreading chlorotic symptoms of DC3000 at similar time points (data not shown). No damage was apparent when adult leaves were infiltrated with *E. coli*. These data indicate that the host damage by *E.coli* is a characteristic of this system and not a general host damage in all conditions.

**Table 1:** Growth of different microbes in the plant growth medium and in the presence of seedlings.

	day 0	day 3		
microbe		medium	conditioned medium	whole well
PA14	5.38	6.9 SD 0.27	7.7 SD 0.39	>9.5
PA14::*lasR*	5.30	ND	ND	>9.5
*B.subtilis*	4.00	4.4 SD 0.5	5.6 SD 0.16	6.6 SD 0.18
*E. coli*	5.62	6.3 SD 0.15	5.35 SD 0.13	7.7 SD 0.3
DC3000	4.84	6.9 SD 0.02	5.04 SD 0.35	>9.5
DC3000/AvrB	4.70	ND	ND	>9.5
DC3000::*hrcC*	4.84	ND	ND	>9.5

Indicated are the growth (log_10_CFU) of the different microbes in the plant growth medium (MS), in the conditioned medium (i.e., seedlings were removed at the time of addition of microbes), or in the presence of seedlings, 3 dpi.

Supporting the conclusion that the interactions between Arabidopsis seedlings and the two known plant pathogens in the seedling assay emulate well studied aspects of host-pathogen interactions are the observations that the bacterial mutants PA14::*lasR* and DC3000::*hrcC,* which are impaired in the synthesis or delivery of a subset of virulence factors, respectively, were attenuated in their ability to inflict host damage in the submered seedling assay. LasR is a key regulatory factor controlling the quorum sensing response in *P. aeruginosa* [11], which in turn affects the production of a variety of virulence factors and have been shown to affect the virulence of PA14 in adult plants, and other hosts [12]. *P. syringae* HrcC is a key structural component of the type III secretion system conserved in a wide range of gram negative pathogens, that delivers a suite of virulence effectors directly into host cells [13]. These data, the data in Table 1 on microbial load during the different interactions tested, and other data presented below also support the notion that the damage is not simply correlated to microbial load.

**Figure 2:**
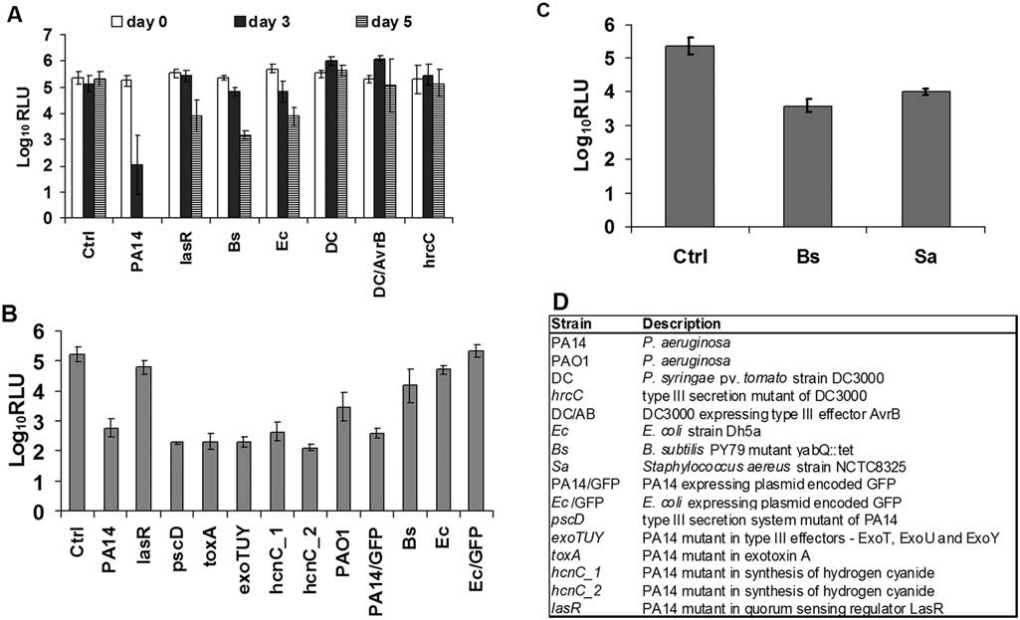
Activity of seedlings engineered to constitutively expressed luciferase after infection with different microbes. (A) The seedlings were infected with microbes as in Fig. 1 and the luciferase activity were measured on the same seedlings before and after addition of microbes (Panel D) at indicated time points. (B) Seedlings were infected with different microbes or different mutants of *P. aeruginosa* strain PA14 (Panel D) and luciferase activity measured 2 dpi. (C) Seedlings were infected with *B. subtilis* or *S. aureus* and luciferase activity measured 3 dpi. Ctrl is control uninfected seedlings. Shown are log_10_ relative luminescence units. (D) List of strains used in the luciferase assays.

In order to have a high-throughput quantitative measure of host damage, we used the activity of a constitutively expressed transgenic luciferase gene [14] as a surrogate of plant health. As can be seen in Fig. 2A, PA14 effectively shuts down the luciferase activity and the activity was completely abrogated by 5 dpi, whereas a PA14 *lasR* mutant only had a modest effect on luciferase activity during this time period. In contrast to PA14, and despite the disease symptoms shown in Fig. 1A, there was no reduction in luciferase activity in seedlings infected with with DC3000. Similar results were obtained with DC3000::*hrcC*, or DC3000 expressing the avirulence gene product AvrB (a type III effector that elicits a potent and rapid cell death response when recognized inside infected cells accompanied by strong resistance, when recognized by a cognate resistance (R) gene product of the host [15], [16]–Fig. 2A. The fact that the highly adapted plant pathogen DC3000 does not cause a reduction in luciferase activity seems counterintuitive, but is consistent with the fact that it is generally considered to be a biotrophic pathogen that depends on its host for nutrition. Biotrophs like DC3000 cause slow host damage to maximize the amount of host-derived nutrition. *B. subtilis* was more effective than *E. coli* in the ability to reduce the luciferase activity, and both were intermediate between PA14 and DC3000. But the fact that DC3000 expressing AvrB, that is expected to elicit an HR and kill the seedlings did not abrogate the luciferase activity in the seedlings and did not seem to reduce the bacterial load significantly (Table 1), is unexpected and highlights unique aspects of this system (discussed later).

The same assay for abrogation of constitutive luciferase activity was used to test the *P. aeruginosa* strain PAO1 [17] and a number of mutants of PA14 affected in known *P. aeruginosa* virulence factors [18]. PAO1 was less potent, than strain PA14. As shown in Fig. 2B, and in contrast to the *lasR* mutant, none of the other tested PA14 mutants were significantly affected in their ability to abrogate the luciferase activity of the seedlings compared to PA14 wild type. Similarly, none of these mutants elicited diminished visual symptoms (not shown). Among the mutants tested were a type III secretion mutant (*pscD*), mutants in three different type III virulence effectors (ExoT, ExoU, and ExoY), an *exoTUY* triple mutant, a mutant in a secreted exotoxin (*toxA*), and two mutants in a gene involved in the production of toxic hydrogen cyanide (*hcnC*). The type III secretion system and the three effectors tested were previously shown to be required for full virulence in insects and mammals [18], but not in *C. elegans* or adult Arabidopsis plants. *hcnC* is a key mediator of toxin-mediated killing in *C. elegans* [19]. ToxA, despite having a minor role in virulence in adult Arabidopsis plants and in a burned mouse model [7], does not have any effect in this system. These data together indicate that the seedling infection system while preserving some well-established aspects of host-pathogen interactions observed in other pathosystems, is unique in some aspects. Thus, the particular features of this infection system fundamentally modifies host and pathogen status such that maladaptive interactions of some microbes that would not normally be initiated are favored.

*E. coli* strain Dh5α harboring a plasmid expressing GFP [8] was much less effective in inflicting host damage (Fig. 1B) and elicited a reduced effect on luciferase activity compared to Dh5α (Fig. 2B). In contrast, when the GFP expressing plasmid was transformed into a different DH5a background, the reconstructed GFP expressing strain was just as virulent as wild-type DH5α. This result implies that the original GFP-expressing strain had likely accumulated a genetic change further supporting the fact that the host damage caused by Dh5α in this system involves an active host-microbe interaction. In addition, PA14 with GFP expressed from a plasmid [8] elicited the same decrease in luciferase activity as PA14 (Fig. 2B), as well as visual symptoms (not shown). In addition, in seedlings treated with the extensively studied laboratory model strain NCTC8325 of the opportunistic gram-positive pathogen *Staphylococcus aureus* [20], luciferase activity was partially abrogated despite the fact that it only elicited very minor disease symptoms (Fig. 1C). Thus, stunting of seedling growth and loss of chlorophyll does not necessarily correlate with seedling health as measured by the constitutive expression of luciferase. Activity of constitutively expressed luciferase or loss of chlorophyll are used as sole indicators for host health/damage in many systems and plants, respectively. The data presented above emphasize the need for multifactorial measurements in the study of host-microbe interactions even in systems with seemingly similar visual phenotypes.

**Figure 3:**
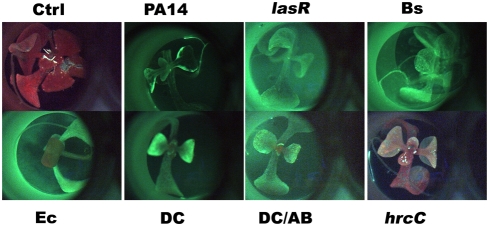
Wide angle micrographs of membrane permeabilization in seedlings infected with different microbes. Wide angle microscopy of seedlings sytox green staining in green fluorescence channel. (18X magnification). Seedlings were stained 3 dpi with membrane impermeable dye Sytox green (SG) and imaged in transparent bottom wells of 96 well plates.

**Figure 4:**
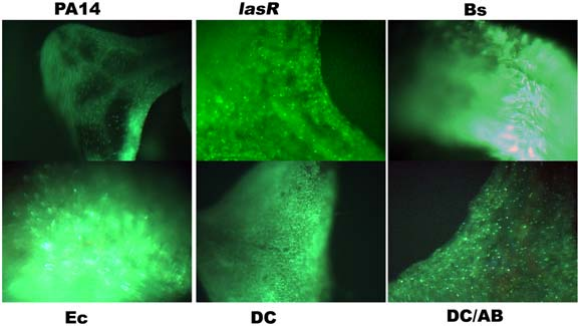
Higher magnification wide angle micrographs of membrane permeabilization in seedlings infected with different microbes. Wide angle microscopy of seedlings sytox green staining in green fluorescence channel. (100X magnification, except PA14 treated seedlings was imaged at 55X magnification). Seedlings were stained 3 dpi with membrane impermeable dye Sytox green (SG) and imaged in transparent bottom wells of 96 well plates.

**Figure 5:**
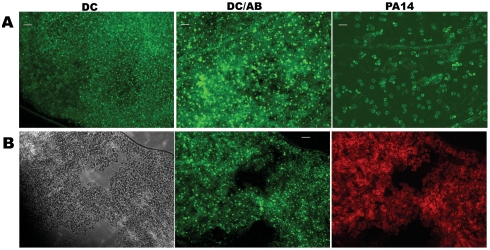
Characteristic cellular damage in seedlings infected with different microbes. (A) Seedlings were stained 3 dpi with membrane impermeable dye Sytox green after treatment with *P. syringae* pv. *tomato* DC3000 (DC), DC3000 expressing the avirulence protein AvrB (DC/AB), and *P. aeruginosa* PA14. (B) Seedlings were stained 3 dpi with membrane impermeable dye Sytox green or the membrane permeable dye Syto 59 after infection with *B. subtilis*, left to right: bright field, Sytox green staining, and Syto 59 staining. Scale bars indicate 50 µm.

To test if the different microbes cause similar histopathological damage in Arabidopsis seedlings, a cell impermeable nucleic acid dye Sytox Green (SG) was used to probe the different interactions. SG enters cells with compromised membrane integrity and fluoresces when interacting with double stranded nucleic acids. Fig. 3 shows green fluorescence due to SG staining of permeabilized cells of Arabidopsis seedlings during interaction with the four different bacterial strains. The control seedlings are bigger and do not exhibit SG staining, whereas the infected seedlings show staining to varying extents. Of particular interest is the fact that seedlings interacting with *B. subtilis* are transparent, indicative of extensive damage and loss of tissue integrity. A slightly higher magnification using wide angle microscopy (Fig. 4) showed a characteristic circular staining pattern on the leaves of seedlings interacting with PA14, more pervasive staining during interaction with PA14::*lasR*, uniform staining of seedlings interacting with DC3000, a large punctuate staining pattern (what appears to be clusters of membrane permeable cells) in seedlings treated with DC3000 expressing AvrB, extremely bright fluorescence in seedlings interacting with *B. subtilis* (indicating extensive damage), and bright fluorescence in patches in seedlings interacting with *E. coli* (Fig. 4). A higher magnification of leaves from seedlings interacting with DC3000 and DC3000 expressing AvrB and PA14 is shown in Fig. 5A. These results indicate that AvrB likely causes permeabilization (probably cell death) in clusters of cells compared to DC3000, despite the fact that in both cases the seedlings do not seem to lose significant luciferase activity. This indicates that while the characteristic cell death response expected of an Avr-R gene interaction is still active, the resistance response that normally accompanies the cell death is not effective. Previous studies have shown that submerged seedlings do express genes strongly associated with disease resistance (e.g., *PR1*) in response to treatment with host and microbe derived defense elicitors (oligogalacturonides and flagellin, respectively) [21]. These data again emphasize the fact that characteristic interaction patterns of known host-pathogen interactions are active in the system (e.g., behavior of *lasR* and *hrcC* mutants, characteristic staining patterns of seedlings treated with different microbes) but the environment-mediated alterations of the host and microbe in the submerged seedling assay favors continued interaction which might otherwise be terminated or severely diminished (e.g., DC3000 expressing AvrB, *E. coli* and *B. subtilis*).

**Figure 6:**
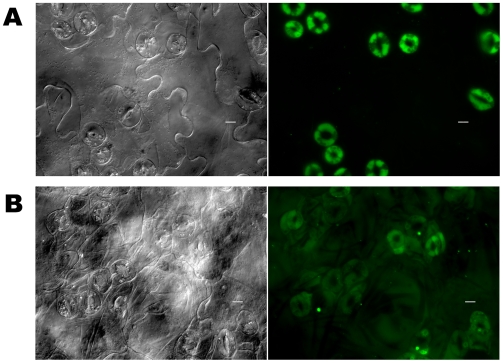
Characteristic changes of seedlings infected with *P. aeruginosa* PA14 or a *lasR* mutant. Seedlings were stained 3 dpi with membrane impermeable dye Sytox green (SG) after infection with (A) *P. aeruginosa* (PA14), (B) PA14::*lasR*. Bright field image on left, sytox green fluorescence on the right. Scale bar indicates 10 µm.

**Figure 7:**
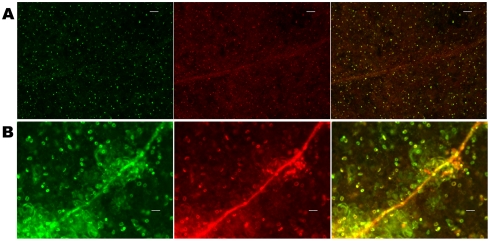
Multiple dyes reveal the same characteristic changes of seedlings infected with *P. aeruginosa* or *P. syringae* pv. *tomato*. Seedlings were stained 3 dpi with membrane impermeable dye Sytox green (SG) in combination with propidium iodide (PI)–another cell impermeant nucleic acid stain after infection with (A) *P. syringae* pv. *tomato* DC3000 (PA14), (B) *P. aeruginosa*. Left: SG staining, middle: PI staining, and right: merge of both. Scale bar indicates 50 µm.

The unusual staining patterns of leaves from seedlings infected with PA14 or *B. subtilis* were further examined at higher magnification (Fig. 5, Fig. 6, Fig. 7). In the case of seedlings interacting with *B. subtilis,* loss of tissue integrity was often seen as big gaps between cells in some locations, corroborating the visually severe damage and strong staining and transparent leaves observed at lower magnifications (Fig. 3D). Many other locations also showed an amorphous mass of cells indicative of loss of tissue integrity. The latter fact is highlighted in micrographs of a leaf from a seedling infected with *B. subtilis* stained with Syto 59, a dye that stains both intact and membrane permeabilized cells (Fig. 5B).

Leaves of seedlings interacting with PA14 showed a very unique pattern of staining of many but not all stomata (Fig. 5A, Fig. 6 and Fig. 7B and consistent with Fig. 2B). Interestingly, this staining was observed throughout the stomatal (guard) cells as opposed to punctuate nuclear staining or diffuse background expected of this class of stain. In order to rule out the possibility that permeant stomatal guard cells were uniformly staining because they had a change in cellular status (e.g., physiological status like redox) specific to SG that increased the stokes shift of green fluorescence in the absence of nucleic acid binding, the seedlings were costained with another nucleic acid staining membrane impermeable dye, propidium iodide (PI). As shown in Fig. 7A seedlings interacting with DC3000 showed staining consistent with the expected pattern of nuclear staining, and the fluorescence from the SG channel (green) and PI channel (red) overlapped. In the case of seedlings interacting with PA14 the staining was uniform over the stomatal (guard) cells and the staining in the SG and PI channels overlapped perfectly (Fig. 7B). These results indicate that the whole cell staining observed in many guard cells is not specific to SG. This raises the intriguing possibility that PA14 invades the stomatal guard cells, as both these dyes can stain permeabilized bacterial cells as well. Typically, bacterial pathogens of plants are intercellular. In the case of PA14 it has earlier been shown by scanning electron microscopy that some bacterial cells seem to punch holes through some plant cell types, and the plant cell walls become ruffled [22]. However, the profile of bacterial cells (unstained and alive i.e., not stained by SG–as in Fig. 6A) as well as observations with GFP expressing PA14 provided no evidence for the presence of live or intact bacteria in guard cells. Similarly, there is no obvious evidence for intact bacteria inside the guard cells in electron micrographs (Fig. S3). Such analysis was not carried out in the case of *B. subtilis*.

In the case of seedlings interacting with the PA14::*lasR* mutant, the staining was pervasive and diffuse in many cell types, but was less intense in the stomatal guard cells (Fig. 6B). This indicates that LasR plays a major role in the stomatal staining phenotype. It can also be inferred that virulence factors produced by PA14 in the presence of functional LasR incites rapid and potent damage to the host. The *lasR* mutant is still able to elicit damage (despite attenuated) and causes stunting of seedlings that differs from wild type PA14 (Fig. 1A, Fig. 4, and Fig. 6B), indicating the presence of additional *lasR* independent factors that by themselves cause this partially impaired and phenotypically different damage (e.g., pervasive staining of many cell types compared to stomatal staining of PA14).

Additional support for the conclusion that the environment of the submerged seedling assay also alters the host in fundamental ways is the fact that often two and sometimes more guard cells could be observed in contact with each other (Fig. 5, Fig. 6A, Fig. 6B and Fig. 7B). This is the case in control (Fig. S4) as well as seedlings infected with various microbes, and violates fundamental rules of normal cell patterning in leaves. Normally, guard cells prevent their neighbors from attaining the same cell fate [23]. A set of LRR containing receptor like kinases, a probable secreted protein ligand, members of a map kinase cascade and myb family transcription factors controlling this patterning and cell fate decision have been identified [24]–[27]. Thus, the patterning defect observed in this submerged seedling system indicates that while the seedlings look normal, overall hormone and other soluble signals or local concentrations that operate in cell-cell communication are impaired in this environment. Such signals that alter host-microbe interactions and defense pathways are likely responsible in part for the differences in responses observed in this system and probably operative in nature.

Earlier it had been shown that in certain assays *E. coli* and *B. subtilis* can elicit characteristic immune gene expression responses in *Drosophila* [28]. Previous studies have also shown an involvement of active defense in *Drosophila* for survival of infection by commonly non-pathogenic *E. coli* [29]. While those studies highlight that these commonly considered non-pathogenic microbes can also infect other hosts also under appropriate conditions, a significant advantage of the system developed here is amenability to high-throughput automation assisted screens for components that perturb these interactions (e.g., genetic and chemical compound screens). Thus these interactions form a metasystem (each host-microbe interaction forming a system) of study that uses simple model host and pathogens and microbes that will recapitulate some aspects of artificial intermingling of organisms that is of health concern in terms of artificial adaptation of microbes to hosts and their ability to cause disease. The host and the microbes used here are extensively studied models with a number of tools and have a large knowledge base and are considered benchmarks to test new theories on; i.e., they are framework model organisms. The study of these interactions and adaptations will provide an excellent framework to build and test these new theories and to evolve newer systems with complementary characteristics. Such an understanding will help identify and avoid the unfavorable conditions that favor host-pathogen adaptations and predict the extent of adaptations in natural settings that have negative impact on mankind. In instances where the knowledge base derived from the model system suggests that the adaptation has progressed extensively, more drastic measures to curb the impact can be initiated.

## Materials and Methods

### Plant growth and microbe inoculation and growth assays

Arabidopsis seeds Columbia (Col) ecotype or seeds of transgenic Col plants expressing humanized Renilla luciferase (hRLUC) under a constitutive dual CaMV 35S promoter [14] were sterilized and stratified at 4°C for three days before use. For experiments with plants grown in soil, the seeds were germinated and grown for the length of time indicated in soil mix (Fafard # 2 mix, Conrad Fafard, USA) and maintained at 23/21°C 12 h day length with 75 µE light and 60% humidity. The different bacteria used were described in references cited. *B. subtilis* used was a sporulation deficient *yabQ*::tet mutant (RL2244) of strain PY79 from Richard Losick (Harvard University) [30]. *E. coli* was Dh5α background. The two *hcnC* mutants of PA14 were NRlib 03_2:e9 and NRlib 15_1:a4 from the transposon insertion library collection [31]. For assays on soil grown seedlings, *P. aeruginosa* strain PA14, *P. syringae* pv. *tomato* strain DC3000, *B. subtilis*, or *E. coli* were sprayed at an ABS_600nm_ of 0.2 (centrifuged cell pellet resuspended in water with 0.01% Silwet L-77-Lehle Seeds, USA) on day 10 and day 12 and the symptom recorded 5 days later. Four week old adult plants were infiltrated with DC3000, *B. subtilis*, *E. coli* or water at 0.02 ABS_600nm_ and symptoms recorded 4 days later.

For assays with liquid seedling the seedlings were germinated in 96 well white wall plates (Cellstar, Greiner bio-one, Germany) in 100 ul MS medium (made from prepackaged powder for 1L from Phytotechnology Laboratories, USA) adjusted to pH 5.8 with 1M KOH and maintained in 22°C for 10 days, at which point they were infected with log phase cultures of appropriate bacteria with 10 µl of 2×10^−4^ ABS_600nm_. Dh5α was used at four times that optical density for Fig. 1B and Table 1, RL2244 was used at twice that (4×10^−4^ ABS_600nm_) optical density for Fig. 2B, Fig. 2C and Table 1. Experiments shown in Fig. 1A and Fig. 2A were in seedlings grown at 40 µE and seedlings for the remaining experiments were grown at 75 µE light. After inoculation the seedlings were maintained at 25°C. The symptoms (visible host damage and abrogation of luciferase activity) progressed faster at 75 µE (3 dpi was approximately equivalent to 5 dpi at 40 µE). The plates were maintained at 80% humidity. For assay of colony forming units (CFUs) of different bacteria, the initial inoculum or the contents of the whole well (after grinding) at indicated time point were diluted appropriately and plated to determine the CFUs. In the case of PA14 and DC3000 and their derivatives the colony count exceeded the countable range of the dilutions plated (i.e., resulted in greater than 9.5 log_10_CFUs per ml).

### Cell staining and microscopy

For staining 1 µM of sytox green, 0.5 µg/mL propidium iodide or 5 µM Syto59 (all from Invitrogen, USA). 10 µl of 10X stock in water were directly added to the wells with seedlings and incubated for 3h to overnight. Wide angle microscopy was done using Discovery V12 microscope (Zeiss, Germany) with Achromat S 1,0X FWD 69 mm lens at 18x or 100x magnification using filter cubes and excitation sources designed for green fluorescence in transparent bottom white wall plates (μclear, CellStar, Greiner bio-one, Germany). Higher magnification microscopy were done using AxioImager 21 microscope (Zeiss, Germany) as DIC, or in epifluorescence mode using excitation laser and filter cubes (Chroma Technology Corporation, USA) designed for either GFP (for green fluorescence–sytox green) or Cy3 (for red fluorescence–propidium iodide and Syto 59).

### Luciferase assays

Luciferase assays were done by adding coelenterazine (Biotium, USA) to the wells with seedlings at a final concentration of 0.5 µM, incubated 20 minutes in dark and luciferase activity measured for 5 secs/well at 20°C and expressed as CPM in the high throughput luminescence reader TopCount NXT (Perkin Elmer, USA).

### Electron Microscopy

Electron microscopy were carried out at the Microscopy Core, Program in Membrane Biology, at the Massachusetts General Hospital. Briefly, medium from seedlings 3 days after treatment with bacteria or untreated control grown in parallel was exchanged with fresh medium. Seedlings were fixed in 2.0% glutaraldehyde in 0.1 M sodium cacodylate (Electron Microscopy Sciences, Hatfield, PA), pH 7.4. They were then rinsed in cacodylate buffer and post-fixed in 1.0% osmium tetroxide (EMS) in 0.1 M cacodylate buffer for one hour at room temperature, and then rinsed as above. After a further rinse in double distilled water, the samples were stained, en bloc in an aqueous solution of 2.0% uranyl acetate for one hour at room temperature. The samples were rinsed in water and dehydrated through a graded ethanol series to 100% ethanol, then infiltrated with EPON (Ted Pell, Inc, Redding, CA)) in a solution of 1:1, EPON: 100% ethanol overnight on a rotator. The next day, they were placed in fresh EPON for several hours and then embedded in EPON overnight in a 60 C oven. Ultrathin sections were cut on a Reichert Ultracut E ultramicrotome. They were collected on formvar-coated grids, stained with lead citrate and uranyl acetate, and examined in a JEOL 1011 transmission electron microscope at 80 kV. Images were acquired using an AMT (Advanced Microscopy Techniques, Danvers, MA) digital imaging system.

## Supporting Information

Figure S1 Symptom development after treatment of soil grown Arabidopsis seedlings treated with *P. syringae* pv. *tomato* strain DC3000. The seedlings were sprayed with water (left panel) or DC3000 (right panel) at 10 and 12 days and symptom recorded 5 days after second spray. Seedlings treated similarly with *P. aeruginosa* PA14, *B. subtilis* or *E. coli* did not show any symptom.
(3.50 MB TIF)

Figure S2 Visual phenotype of adult leaves of Arabidopsis infiltrated with *B. subtilis* or *E. coli*. Leaves of 4 week old soil grown plants were infiltrated with bacteria at ABS_600nm_ of 0.02 and symptoms recorded 4 dpi.
(2.37 MB TIF)

Figure S3 Impaired guard cell patterning on leaves of liquid grown seedlings. Shown are three examples (DIC micrographs) of leaves from a 13 day old control untreated seedlings and a PA14 treated seedling, 3dpi infected on day 10, (bottom right) to highlight impairment in guard cell patterning and specification. All frames were taken at same magnification. Scale bar (upper left) represent 10 µm.
(8.36 MB TIF)

Figure S4 Electron micrographs from liquid grown seedlings treated with *P. aeruginosa* PA14 do not show intact bacterial cells inside the guard cells. Leaves of liquid grown seedlings treated with PA14 (on day 10) were subjected to transmission electron microscopy 3 dpi. Three pairs of guard cells from PA14 treated seedlings are shown (panels A–C), because many and not all guard cells show the characteristic staining phenotype upon infection with PA14. (D) and (E) indicate higher magnification of the regions indicated by squares on the left and right in panel (C), respectively. Representative mitochondria indicated by the letter m beside them, bacteria indicated by letter b beside it, regions outside the plant leaf surface are indicated by letter e, and guard cells indicated by letter g inside. Scale bars represent 2 µm in panels A–C, and 500 nm in panels D and E.
(1.62 MB TIF)
