# Supplementary figures and images for: A Metasystem of Framework Model Organisms to Study Emergence of New Host-Microbe Adaptations
Source: PLoS One. 2008 Dec 10;3(12):e3891. doi: 10.1371/journal.pone.0003891 (PMC2588659; doi:10.1371/journal.pone.0003891)

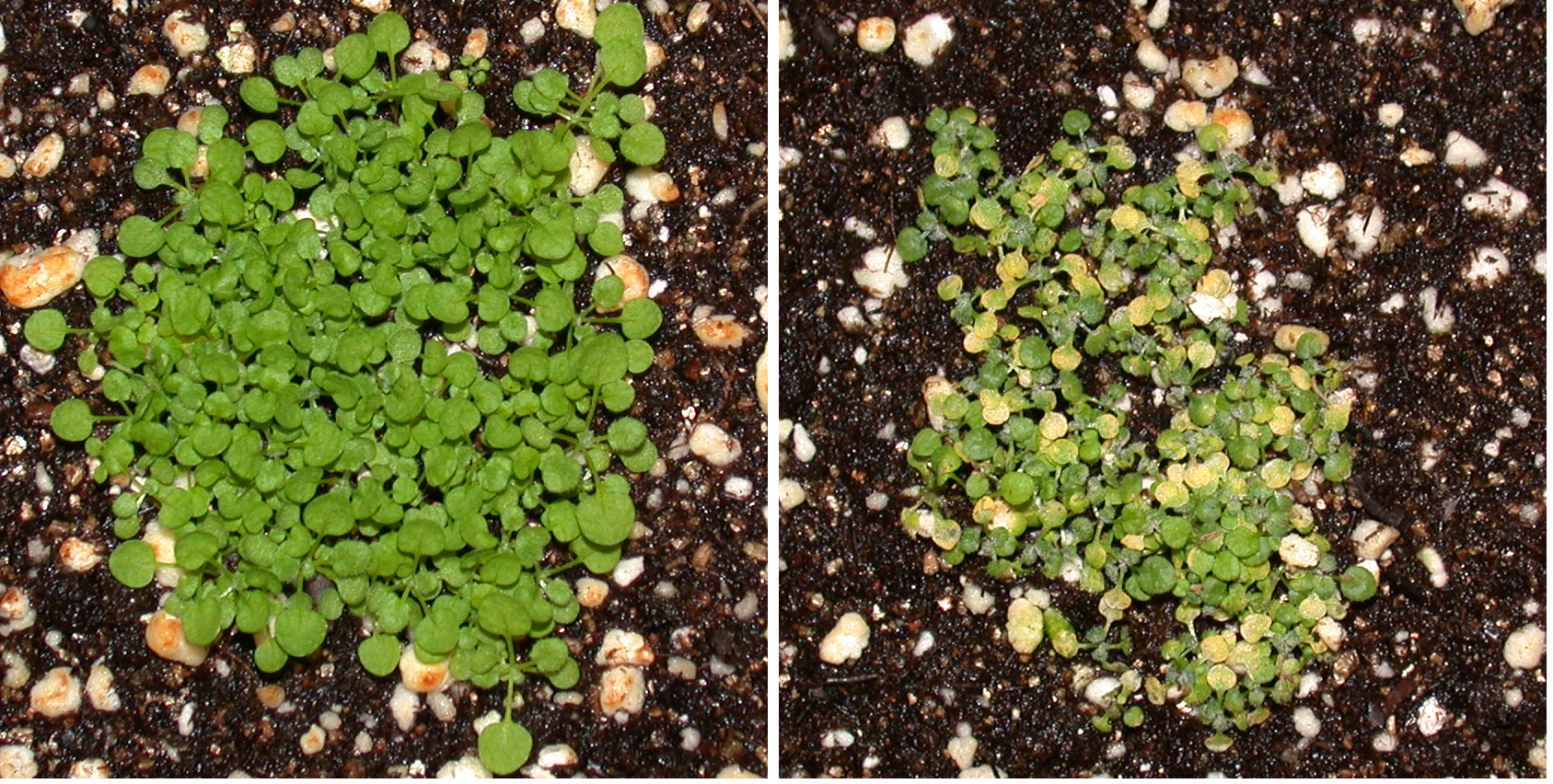

Supplement: Figure S1 — Symptom development after treatment of soil grown Arabidopsis seedlings treated with P. syringae pv. tomato strain DC3000. The seedlings were sprayed with water (left panel) or DC3000 (right panel) at 10 and 12 days and symptom recorded 5 days after second spray. Seedlings treated similarly with P. aeruginosa PA14, B. subtilis or E. coli did not show any symptom. (3.50 MB TIF) [file pone.0003891.s001.tif]

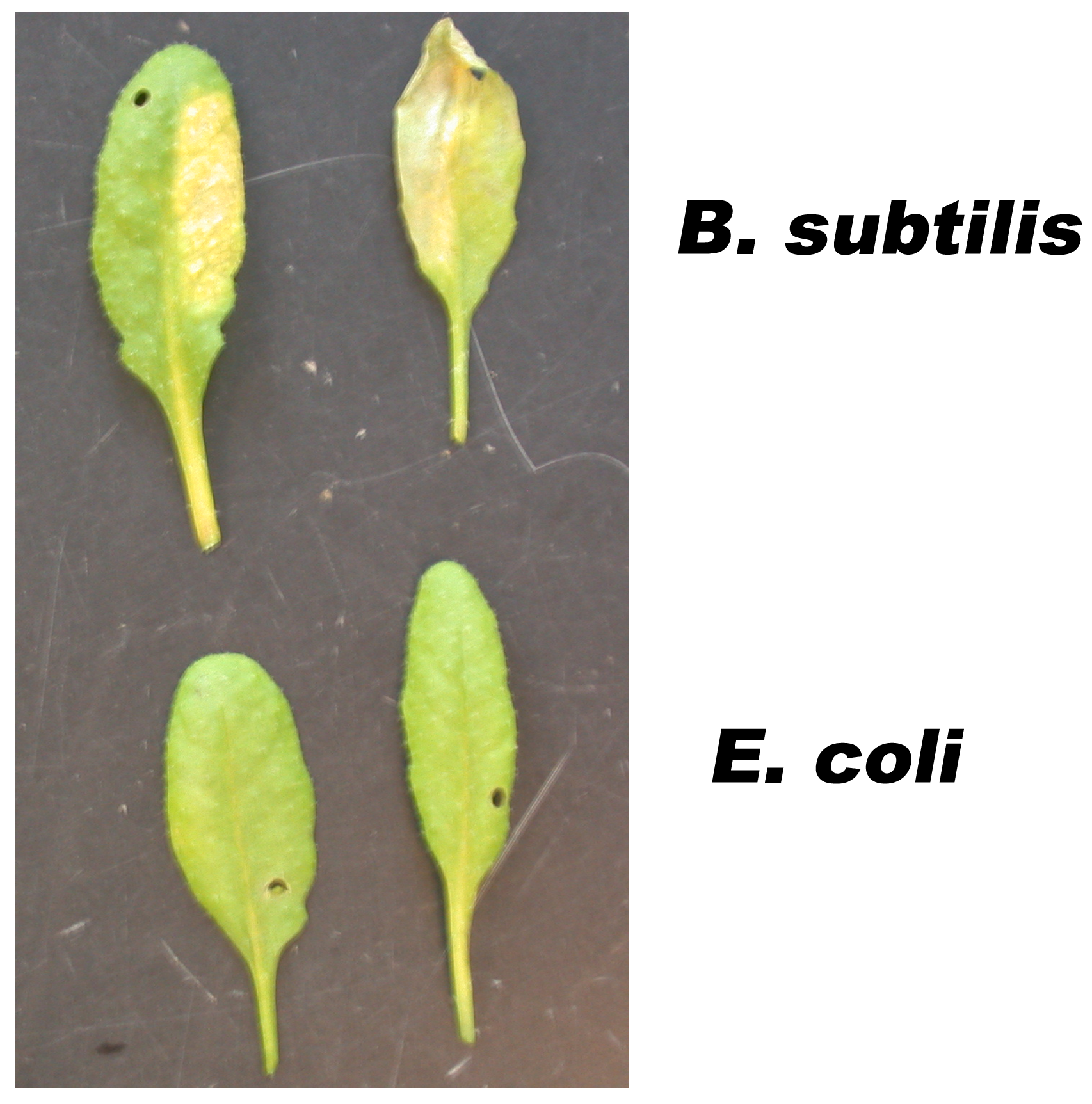

Supplement: Figure S2 — Visual phenotype of adult leaves of Arabidopsis infiltrated with B. subtilis or E. coli. Leaves of 4 week old soil grown plants were infiltrated with bacteria at ABS600nm of 0.02 and symptoms recorded 4 dpi. (2.37 MB TIF) [file pone.0003891.s002.tif]

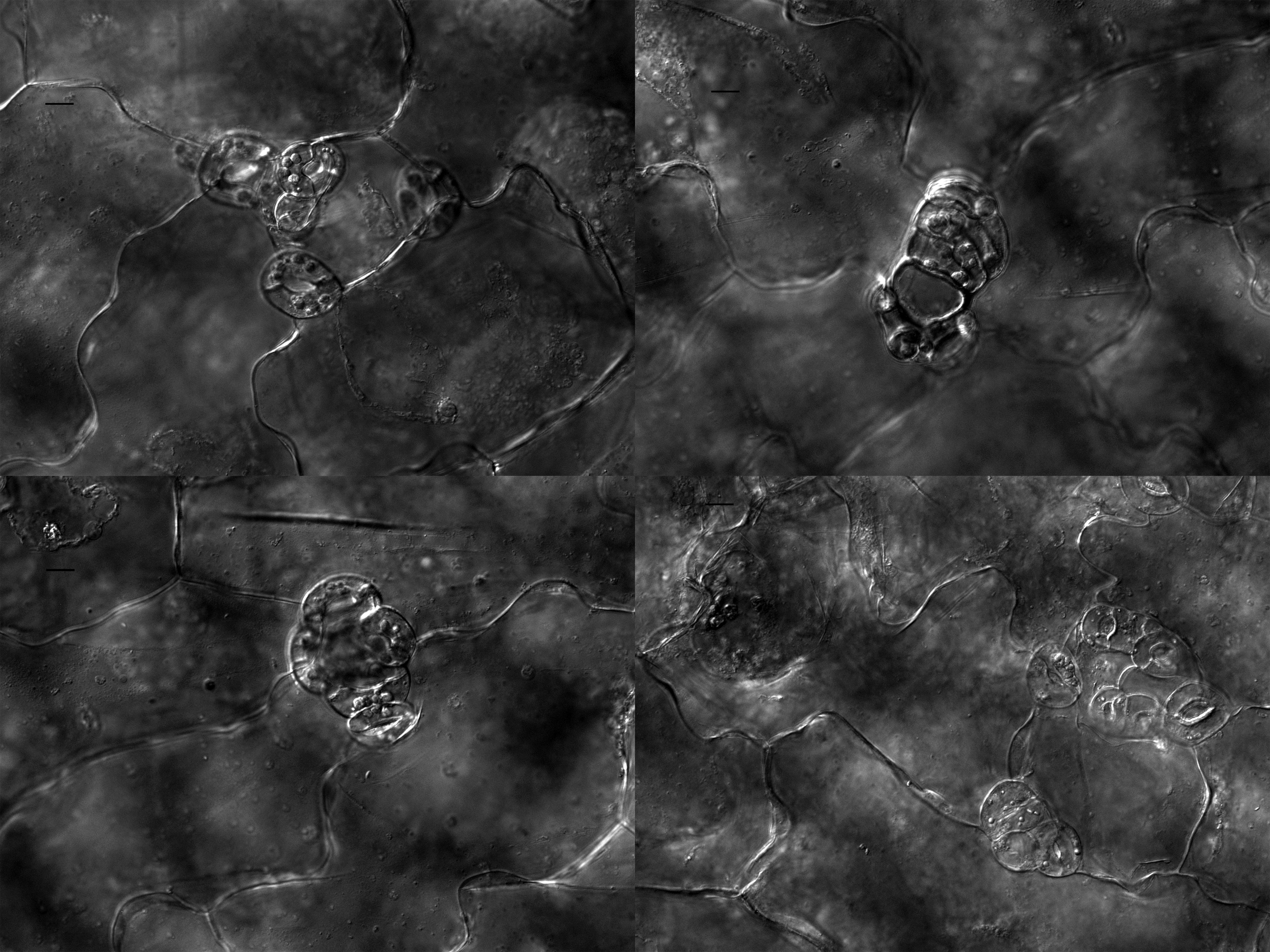

Supplement: Figure S3 — Impaired guard cell patterning on leaves of liquid grown seedlings. Shown are three examples (DIC micrographs) of leaves from a 13 day old control untreated seedlings and a PA14 treated seedling, 3dpi infected on day 10, (bottom right) to highlight impairment in guard cell patterning and specification. All frames were taken at same magnification. Scale bar (upper left) represent 10 µm. (8.36 MB TIF) [file pone.0003891.s003.tif]

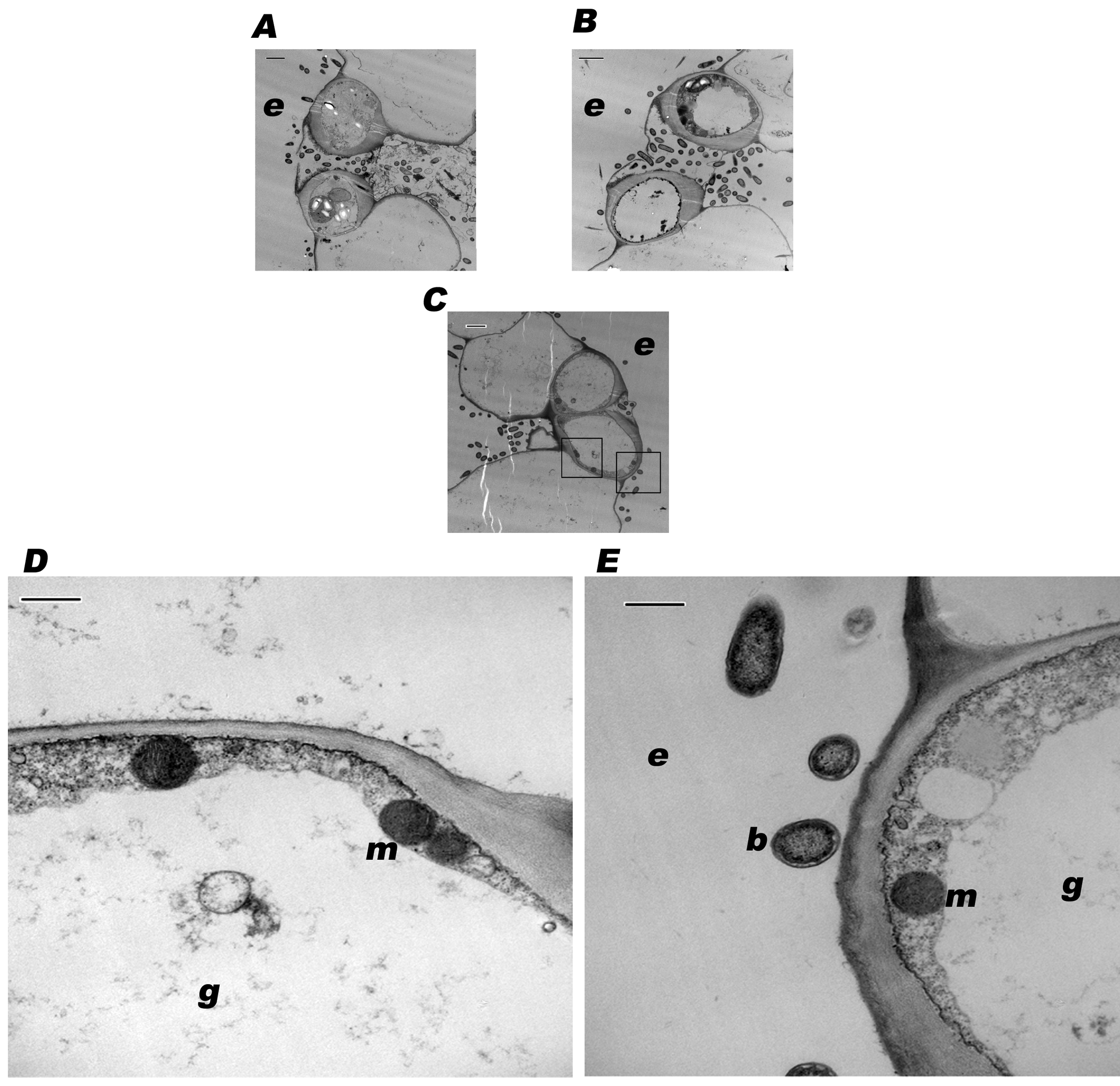

Supplement: Figure S4 — Electron micrographs from liquid grown seedlings treated with P. aeruginosa PA14 do not show intact bacterial cells inside the guard cells. Leaves of liquid grown seedlings treated with PA14 (on day 10) were subjected to transmission electron microscopy 3 dpi. Three pairs of guard cells from PA14 treated seedlings are shown (panels A–C), because many and not all guard cells show the characteristic staining phenotype upon infection with PA14. (D) and (E) indicate higher magnification of the regions indicated by squares on the left and right in panel (C), respectively. Representative mitochondria indicated by the letter m beside them, bacteria indicated by letter b beside it, regions outside the plant leaf surface are indicated by letter e, and guard cells indicated by letter g inside. Scale bars represent 2 µm in panels A–C, and 500 nm in panels D and E. (1.62 MB TIF) [file pone.0003891.s004.tif]
